# Supplementary material for: Diagnostic and prognostic implications of growth differentiation factor 15 in heart failure with preserved ejection fraction: a systematic review and meta-analysis
Source: PeerJ. 2025 Oct 28;13:e20168. doi: 10.7717/peerj.20168 (PMC12577569; doi:10.7717/peerj.20168)
Supplement: Supplemental Information 2 [file peerj-13-20168-s002.docx]

**SUPPLEMENTARY MATERIALS**

**Supplementary Table 1.** Risk of bias analysis for the included studies, according to the Newcastle-Ottawa quality and Newcastle-Ottawa adapted for cross-sectional study assessment scale

| **Study** | **Selection** | | | | **Comparability** | **Outcome/Exposure** | | | **Total Score** |
| --- | --- | --- | --- | --- | --- | --- | --- | --- | --- |
|  | **1**  **(Representativeness of the exposed cohort)** | **2**  **(Selection of non-exposed cohort)** | **3 (Ascertainment of exposure)** | **4 (Demonstration that outcome of the interest was not present at the start of the study)** | **1 (Comparability of cohorts on the basis of the design or analysis)** | **1**  **(Assessment of outcome)** | **2**  **(Follow-up long enough for outcomes to occur)** | **3 (Adequacy of follow-up of cohort)** |  |
| Baessler et al, 2012 | 1 | 1 | 1 | 1 | 2 | 1 | 0 | 0 | 7 |
| Santhanakrishnan et al, 2012 | 1 | 1 | 1 | 1 | 2 | 1 | 0 | 0 | 7 |
| Izumiya et al, 2014 | 1 | 1 | 1 | 1 | 1 | 1 | 1 | 1 | 8 |
| Sinning et al, 2017 | 1 | 1 | 1 | 1 | 2 | 1 | 1 | 1 | 9 |
| Kanagala et al, 2020 | 1 | 0 | 1 | 1 | 2 | 1 | 1 | 1 | 8 |
| Jirak et al, 2020 | 1 | 1 | 1 | 1 | 1 | 1 | 0 | 0 | 6 |
| Mendez Fernandez et al, 2020 | 1 | 1 | 1 | 1 | 1 | 1 | 1 | 1 | 8 |
| Aulin et al, 2022 | 1 | 1 | 1 | 1 | 2 | 1 | 1 | 1 | 9 |
| Yin et al, 2023 | 1 | 1 | 1 | 0 | 2 | 1 | 1 | 1 | 8 |
| Oyama et al, 2023 | 1 | 1 | 1 | 1 | 2 | 1 | 1 | 1 | 9 |
| Lyu et al, 2024 | 1 | 1 | 1 | 0 | 2 | 1 | 1 | 1 | 8 |
|  |  |  |  |  |  |  |  |  |  |
| **Study** | **1  (Representativeness of the sample** | **2  (Sample size)** | **3  (Non-respondents)** | **4  (Ascertainment of the exposure risk factor)** | **1 (Comparability analysis)** | **1  (Assessment of outcome)** | **2   (Statistical test)** | **Total** |  |
| Mitic et al, 2020 | 1 | 1 | 1 | 2 | 2 | 2 | 1 | 10 |  |

**Supplementary Table 2.** Baseline data comparing GDF-15 and NT-proBNP in HFpEF and control group

| **Study** | **HFpEF** | | | | **Control** | | | |
| --- | --- | --- | --- | --- | --- | --- | --- | --- |
|  | **Sample size (n)** | **LVEF (mean ± SD / median (IQR)) (%)** | **GDF-15 (mean ± SD / median (IQR)) unit** | **NT-proBNP (mean ± SD / median (IQR)) unit** | **Sample size (n)** | **Definition of control group** | **GDF-15 (mean ± SD / median (IQR)) unit** | **NT-proBNP (mean ± SD / median (IQR)) unit** |
| Baessler et al. 2012 | 88 | 64.0 ± 9.0 | 665.0 (496.0–926.0) pg/mL | 52.0 (29.0–96.0) ng/L | 119 | Obese with normal LV function | 451.0 (392.0–679.0) pg/mL | 42.0 (25.0–66.0) ng/L |
| Lyu et al. 2024 | 322 | 57.0 (53.0–60.0) | 2921.2 (1865.8–5057.0) ng/L | 673.5 (304.5–1,896.5) pg/mL | - | | | |
| Sinning et al. 2017 | 70 | 63.9 (58.9-69.5) | 1290.0 (989.3-1868.8) ng/L | 145.5 (75.5, 293.9) pg/mL | 4864 | Non-HF population | 885.0 (725.9-1136.0) ng/L | 61.9 (28.5-124.4) pg/mL |
| Aulin et al. 2022 | 2520 | 56.0 (50.0-62.0) | 1409.0 (970.0-2,091.0) ng/L | 791.0 (417.0 - 1371.2) ng/L | 7250 | AF without history of HF | 1328.0 (957.2-1930.0) ng/L | 615.0 (312.0-1,069.5) ng/L |
| Oyama et al. 2023 | 2016 | - | 1599.0 (1115.0-2344.0) pg/mL | 782.0 (367.0-1425.0) pg/mL | 3530 | AF without history of HF | 1674.0 (1237.0-2406.0) pg/mL | 678.0 (296.0-1182.0) pg/mL |
| Santhanakrishnan et al. 2012 | 50 | 60.0 ± 7.0 | 2528.9 (1247.1-4349.3) pg/mL | 942.0 (309.0-2768.0) pg/mL | 50 | Without coronary artery disease or HF by history, and clinical and echocardiographic examination. | 540.1 (421.2-840.1) pg/mL | 69.0 (41.0-102.0) pg/mL |

**Supplementary Table 3.** Baseline data comparing GDF-15 and BNP in HFpEF and control group

| **Study** | **HFpEF** | | | | **Control** | | | |
| --- | --- | --- | --- | --- | --- | --- | --- | --- |
|  | **Sample size (n)** | **LVEF (mean ± SD / median (IQR)) (%)** | **GDF-15 (mean ± SD / median (IQR)) unit** | **BNP (mean ± SD / median (IQR)) unit** | **Sample size (n)** | **Definition of control group** | **GDF-15 (mean ± SD / median (IQR)) unit** | **BNP (mean ± SD / median (IQR)) unit** |
| Kanagala et al. 2020 | 140 | 56.0 ± 5.0 | 2248.0 (1546.0–3585.0) ng/mL | 136.0 (66.0–254.0) ng/L | 48 | Asymptomatic hypertensive or non-hypertensive controls. | 955.0 (665.0–1300.0) ng/mL | 33.0 (24.0–44.0) ng/L |
| Jirak et al. 2020 | 18 | 59.7 ± 9.8 | 838.0 (1045.9-630.0) pg/mL | 165.22 ± 162.54 pg/mL | 95 | Without HFpEF, DCM, and ICM | 561.2 (422.9-699.5) pg/mL | 73.74 ± 86.08 pg/mL |
| Mitic et al. 2020 | 26 | 53.6 ± 3.7 | 1,493.1 ± 421.4 pg/mL | 92.0 ± 18.9 pg/mL | 35 | Healthy volunteers who were age and gender matched with the selected patients and were without any history of coronary artery disease or HF. | 542.6 ± 48.2 pg/mL | 14.8 ± 7.2 pg/mL |

**Supplementary Table 4.** Diagnostic value of GDF-15 in identifying HFpEF from controls compared to conventional biomarker

| **Study** | **GDF-15** | **Conventional biomarkers** | |
| --- | --- | --- | --- |
|  | **AUC** | **AUC** | **Biomarker** |
| Baessler et al. 2012 | 0.74 | 0.56 | NT-proBNP |
| Sinning et al. 2017 | 0.766 | 0.735 | NT-proBNP |
| Jirak et al. 2020 | 0.787 | - | - |
| Santhanakrishnan et al. 2012 | 0.936 | 0.934 | NT-proBNP |

**Supplementary Table 5.** Multivariate analysis in the prediction of all-cause mortality

| **All-Cause Mortality** | | | | | | | |
| --- | --- | --- | --- | --- | --- | --- | --- |
| **Study** | **HFpEF patients (n)** | **Follow-up duration (months)** | **HR** | **95% Upper CI** | **95% Lower CI** | **P** | **Variables adjusted** |
| Lyu et al, 2024 | 322 | 112 | 1.33 | 1.62 | 1.09 | - | Heart rate; MAGGIC risk score; hypertension; previous myocardial infarction; previous PCI/CABG; stroke; chronic kidney disease; anemia; atrial fibrillation; statin; diuretic; spironolactone; digoxin; calcium channel blocker; creatinine; LDL-C; triglycerides; NT-proBNP, hs-TnT. |
| Sinning et al, 2017 | 70 | 87 | 1.5 | 1.7 | 1.3 | < 0.001 | Cardiovascular risk factors and renal function |
| Yin et al, 2023 | 380 | 12 | 1.9 | 3.68 | 0.98 | 0.057 | ASCEND-HF risk score, history of HF, N-terminal pro-brain natriuretic peptide, and high-sensitivity cardiac troponin T. |
| Mendez Fernandez et al, 2020 | 221 | 15 | 1.008 | 1.013 | 1.003 | 0.01 | - |

**Supplementary Table 6.** Multivariate analysis in the prediction of adverse cardiovascular events

| **Heart Failure Hospitalisation** | | | | | | | |
| --- | --- | --- | --- | --- | --- | --- | --- |
| **Study** | **HFpEF patients (n)** | **Follow-up duration (months)** | **HR** | **95% Upper CI** | **95% Lower CI** | **P** | **Variables adjusted** |
| Izumiya et al, 2014 | 73 | 24 | 4.74 | 17.88 | 1.26 | 0.022 | - |
| Oyama et al, 2023 | 2016 | 33 | 1.98 (RR) | 2.35 | 1.67 | <0.001 | Age, sex, race, body mass index (BMI), history of hypertension, history of diabetes, estimated glomerular filtration rate (eGFR), history of HF, history of myocardial infarction, and pattern of AF (paroxysmal, persistent, or permanent) |

**Supplementary Table 7.** Multivariate analysis in the prediction of heart failure hospitalisation

| **Heart Failure Hospitalisation** | | | | | | | |
| --- | --- | --- | --- | --- | --- | --- | --- |
| **Study** | **HFpEF patients (n)** | **Follow-up duration (months)** | **HR** | **95% Upper CI** | **95% Lower CI** | **P** | **Variables adjusted** |
| Lyu et al, 2024 | 322 | 112 | 1.72 | 2.06 | 1.43 | - | Heart rate; MAGGIC risk score; hypertension; previous myocardial infarction; previous PCI/CABG; stroke; chronic kidney disease; anemia; atrial fibrillation; statin; diuretic; spironolactone; digoxin; calcium channel blocker; creatinine; LDL-C; triglycerides; NT-proBNP, hs-TnT. |
| Yin et al, 2023 | 380 | 12 | 2.25 | 1.43 | 3.54 | <0.001 | ASCEND-HF risk score, history of HF, N-terminal pro-brain natriuretic peptide, and high-sensitivity cardiac troponin T. |
