# Supplementary material for: Diagnostic and prognostic implications of growth differentiation factor 15 in heart failure with preserved ejection fraction: a systematic review and meta-analysis
Source: PeerJ. 2025 Oct 28;13:e20168. doi: 10.7717/peerj.20168 (PMC12577569; doi:10.7717/peerj.20168)
Supplement: Supplemental Information 3 [file peerj-13-20168-s003.docx]

**Audience Intended For**

This systematic review and meta-analysis on the diagnostic and prognostic implications of Growth Differentiation Factor-15 (GDF-15) in Heart Failure with preserved Ejection Fraction (HFpEF) is intended for the following audiences:

1. **Cardiologists and Heart Failure Specialists** – to support evidence-based decision-making regarding the use of GDF-15 as a biomarker for diagnosis, risk stratification, and prognosis in patients with HFpEF.
2. **Clinical Researchers in Cardiovascular Biomarkers** – to provide a comprehensive synthesis of current data, identify existing gaps, and encourage further research into the clinical utility of GDF-15 in HFpEF.
3. **Internal Medicine Physicians and Primary Care Providers** – who are often the first to encounter patients with nonspecific symptoms of HFpEF, this review offers insights into potential biomarker-guided approaches to early detection and management.
4. **Guideline Committees and Health Policy Makers** – to inform the incorporation of emerging biomarkers like GDF-15 into HFpEF clinical practice guidelines and improve standardized care pathways.
5. **Medical Educators, Fellows, and Trainees** – to enhance the understanding of biomarker science, especially in the growing and complex area of HFpEF, and to support learning through evidence-based summaries.
6. **Multidisciplinary HFpEF Care Teams** – including nurses, clinical pharmacists, and allied health professionals, by offering knowledge that can be integrated into holistic and personalized care strategies.
